# Isolated colostomy site recurrence in rectal cancer-two cases with review of literature

**DOI:** 10.1186/1477-7819-5-52

**Published:** 2007-05-13

**Authors:** Chintamani, Vinay Singhal, Anju Bansal, Dinesh Bhatnagar, Sunita Saxena

**Affiliations:** 1 Department of Surgery, Vardhman Mahavir Medical College, Safdarjang Hospital, New, Delhi, India; 2 Institute of Pathology, Indian council of Medical Research, Vardhman Mahavir Medical College, Safdarjang Hospital, New Delhi, India; 3 Vardhman Mahavir Medical College Safdarjang Hospital, New Delhi, India

## Abstract

**Background:**

Colostomy site carcinomas are rare with only eight cases reported in the world literature. Various etiological factors like adenoma-cancer sequence, bile acids, recurrent and persistent physical damage at the colostomy site by faecal matter due to associated stomal stenosis have been considered responsible. Two such cases are being reported and in both cases there was no evidence of any local recurrence in the pelvis or liver and distant metastasis. Both patients had received adjuvant chemotherapy following surgery.

**Case presentation:**

First case was a 30-year-old male that had reported with large bowel obstruction due to an obstructing ulcero-proliferative growth (poorly differentiated adenocarcinoma) at the colostomy site after abdomino-perineal resection, performed for low rectal cancer six years previously. Wide local excision with microscopically free margins was performed with a satisfactory outcome. Four years later he presented with massive malignant ascites, cachexia and multiple liver metastasis and succumbed to his disease.

Second case was a 47-year-old male that presented with acute large bowel obstruction due to an annular growth (well differentiated adenocarcinoma) in the upper rectum. He was managed by Hartmann's operation and the sigmoid colostomy was closed six months later. Five years following closure of colostomy, he presented with two parietal masses at the previous colostomy site scar, which, on fine needle aspiration cytology were found to be well-differentiated adenocarcinomas of colorectal type. Surgery in the form of wide local resection with free margins was performed. He presented again after five years with recurrence along the previous surgery scar and an incisional hernia and was managed by wide local excision along with hernioplasty. Follow-up of nine years following first surgery is satisfactory.

**Conclusion:**

Colostomy site/scar recurrence of rectal carcinoma is rare and could be due to various etiological factors, although the exact causative mechanism is not known. Surgery with microscopically free margins is recommended in the absence of metastatic disease. Stenosis of the stoma is considered as one of the most important contributory factors and should be followed carefully.

## Background

Metachronous carcinomas rarely occur at the colostomy site and only eight cases have been reported previously. Various factors like adenoma-cancer sequence, stenosing stoma or bile acids have been implicated. Colon cancer presenting as cutaneous metastasis in an old operative scar has also been reported [1-6]. Possible etiological factors include an alteration in the microscopic anatomy around the scar, perhaps in the lymphatic channels, altered adhesion molecule profile or altered local immunosurveillance mechanisms leading to change in the local environs of the scar which become more receptive to metastatic tumor cells [7]. The occurrence being so rare, no definite etiology and management protocol is known. Management in the form of curative surgery along with adjuvant chemotherapy is recommended.

## Case presentation

### Case-1

**Figure 1:**
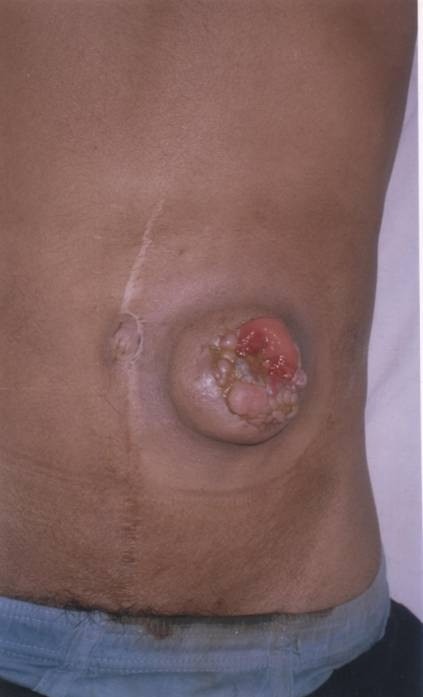
Clinical photograph showing ulceroproliferative growth at the colostomy site. Well-healed midline scar of the previous surgery (abdomino-perineal resection) may be seen.

**Figure 2:**
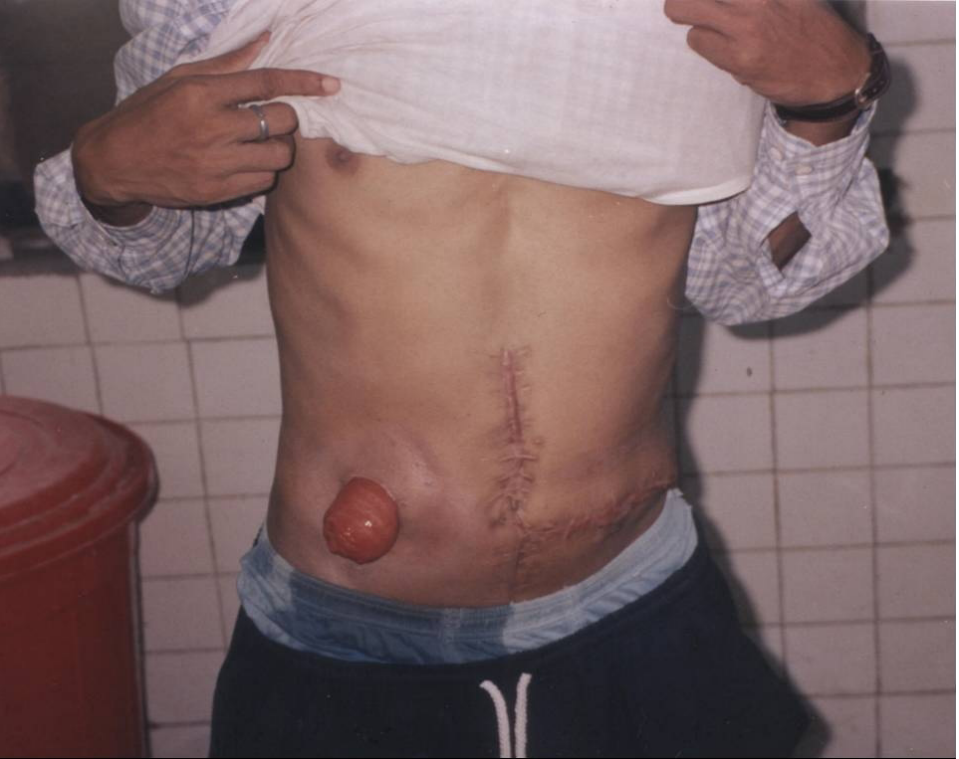
Eight months after second surgery with transverse colostomy on the right side. The well-healed transverse scar on left side of abdomen may be seen.

A-30-year old male had been operated in March 1996 for low rectal cancer (T4N0M0) in the form of classical abdomino-perineal resection with microscopically free (R0) margins and satisfactory outcome. He was on a regular follow-up for three years and was subsequently lost to follow-up. In April 2002 he presented with acute large bowel obstruction due to complete stenosis and infiltration of the stoma site by an ulcero-proliferative growth, which was extending in to the surrounding skin and the abdominal muscles (figure 1). He had been having increasing narrowing of the stoma for the past one year and was managed by his family doctor by regular finger dilatations. Biopsy from the growth was suggestive of poorly differentiated adenocarcinoma. The ultrasound and computed tomography scan ruled out any local recurrence in the pelvis or metastases in the liver or ascites. Blood biochemistry including liver function tests was within normal limits except for anaemia (Hb = 10 gm%) and raised carcino-embryonic antigen (CEA = 10.7 ng/ml, normal being up to 2.5 ng/ml). He was managed by wide local excision including the stoma with wide margin, the entire descending colon, 2/3^rd ^of the transverse colon *en bloc *with the involved para and pre-aortic lymph nodes. The left ureter, which was encased within the lymph node mass, was dissected free and right-sided transverse colostomy was done (figure 2). The histopathological examination revealed poorly differentiated adenocarcinoma of the rectum with signet cell appearance. Out of ten lymph nodes resected along with the specimen, three showed metastasis (rpT4N1M0).

Postoperative recovery was good and he was discharged on the tenth day. He received the adjuvant chemotherapy in the form of Levamisole and 5 fluorouracil three weekly (total 12 cycles). He was again lost to follow-up and presented subsequently in August 2006 with cachexia, massive malignant ascites, and liver metastasis and succumbed to his disease.

### Case.2

**Figure 3:**
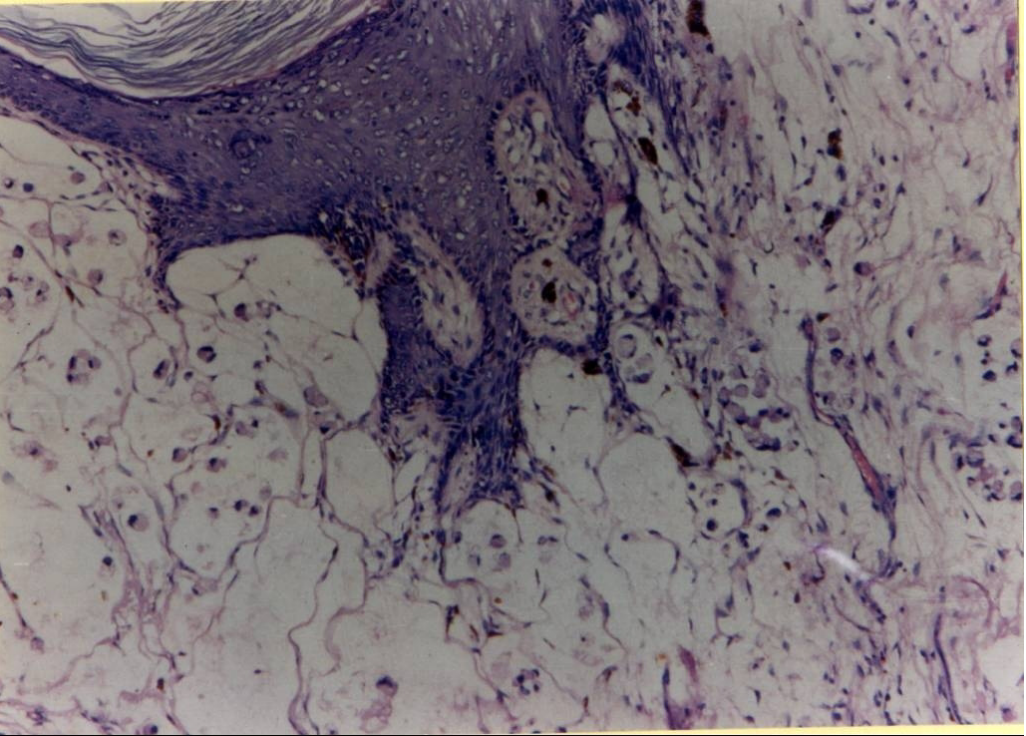
The histopathological examination of the resected recurrent colostomy site specimen showing well-differentiated adenocarcinoma (40×).

A-45-year old male presented to the surgical emergency in Aug 1997 with features of acute large bowel obstruction of three days duration due to an annular growth in the upper rectum without any evidence of liver or peritoneal metastasis. Due to poor general condition (anemia, deranged electrolytes) and the unprepared bowel, he was managed by Hartmann's operation. The histopathological examination of the resected specimen revealed well-differentiated adenocarcinoma of the rectum (T4N1M0). The colostomy was closed after eight months and the regular follow-up was satisfactory. He received adjuvant chemotherapy in the form of Levamisol and 5 fluorouracil. He was subsequently lost to follow-up and reported back five years later in 2002 with two parietal lumps in the left lumbar region and the fine needle aspiration cytology showed them to be well-differentiated adenocarcinoma of colorectal variety. The Ultrasound and contrast enhanced computed tomographic scan (CECT) showed infiltration in to the muscles of the anterior abdominal muscles and the peritoneum. A wide local excision including the abdominal muscles was done and the wound was closed in layers. He reported back in May 2005 with a recurrent 2 × 3 cm parietal nodule along the scar of the previous colostomy and an incisional hernia. The scar recurrence was excised with an R0 margin and the hernia was repaired using an onlay prolene mesh. The histopathological examination of the specimen revealed well-differentiated adenocarcinoma of colorectal type (figure 3). The postoperative recovery was good. He was discharged on the seventh day and a regular follow up of one year (nine years following first surgery) is satisfactory.

## Discussion

Colon is not an uncommon site for synchronous and metachronous malignancies. The criteria for multiple carcinomas of the colon are well laid out [6]. In a study, multiple carcinomas accounted for 4.3% of all colorectal carcinomas, and for 12.9% of colorectal carcinomas and polypoid cancers of the colon (synchronous 4%; metachronous 2.0%) [6-8].

However, adenocarcinoma occurring at the colostomy site with no recurrence in the pelvis is very rare. Only eight similar cases have been reported previously (table 1). There have been case reports of adenoma carcinoma sequence [2,3,5]. The stoma is more easily damaged than the mucosa of the intestine because it may be exposed to unexpected compression by clothing. Narrowing of the stoma or stenosis due to persistent pressure of tight clothing on the stoma has also been found to be a significant contributory factor and such patients should be carefully followed. Similar finding was observed in the second reported case. Cancer family syndrome has also been suggested as a contributory factor by some authors on the basis of positive family history [4]. Others have however argued that because there were no signs of adenoma of the stoma that could be observed on a daily basis, the carcinomas might have been similar to *de novo *metaplasia [1].

**Table 1:** Cases reported in the literature earlier with two present cases

**Investigator**	**Year of report**	**Past history (Age of first surgery in years)**	**Age at presentation with colostomy site adenocarcinoma(years)**	**Sex**	**Time of development of carcinoma at the colostomy site**
Takami et al. [3]	1983	Rectal cancer(19)	38	M	19 years after APR
Saegusa [10]	1986	Rectal cancer(55)	60	M	5 years after APR
Nakano et al. [1]	1987	Rectal cancer(31)	53	F	22 years after APR
Takeyuchi et al. [4]	1990	Rectal cancer(44)	56	M	12 Year after APR
Ohta et al. [2]	1991	Rectal cancer(68)	77	F	9 Years after APR
Ishikawa et al. [1]	1994	Rectal cancer(46)	76	F	30 Years after APR
Ohtsuka et al. [5]	1996	Rectal cancer(77)	81	M	4 Years after APR
Shibuya et al. [1]	1997	Rectal cancer(73)	81	M	8 Years after APR
Our patient case-1	2006	Rectal cancer (30)	36	M	6 years after APR
Our patient case-2	2006	Rectal cancer(45)	50	M	5 years after LAR

APR: Abdominoperineal resection

LAR: Low anterior resection with Hatmann's operation

Presence of enterobacteria and bile acids in the stools has also been implicated and reported and the carcinomas occurring at a stoma with which stools were briefly in contact appeared to be rare [2]. Amongst the eight known cases (table 1), three cases including one of the two reported cases may have been affected by bile acids and changes in enterobacteria that resulted from prolonged contact with stools.

There could be a possible similarity in the mechanisms involved in the recurrence at colostomy site to that of recurrence of cancer in a scar of previous surgery and various causative mechanisms have been suggested. There may be a direct extension of the disease, hematogenous spread, lymphatic spread or implantation of exfoliated tumor cells if the specimen had been retrieved through the incision [7]. This is however unlikely to be the scenario in both the presented cases particularly in the first case with recurrence occurring at the site of colostomy. In the second case, the recurrence developed along the scar of previously performed colostomy and this case could possibly resemble in presentation to an incisional scar recurrence in some ways. Predilection to metastasis at incision site has also been postulated to be due to alteration in the microscopic anatomy around the scar, perhaps in the lymphatic channels, making it more receptive to metastatic tumor cells, possibly due to altered adhesion molecule profile or altered local immunosurveillance mechanisms. The exact pathogenesis however remains a matter of speculation awaiting further recognition and investigation [1,7-10].

## Conclusion

Carcinomas may occur coincidentally at a stoma, but the stricture of the stoma and the physical stimulation like regular pressure of tight clothing, could promote their occurrence. The stricture of a stoma should be followed carefully and patient informed about the possibility of development of carcinoma at the colostomy site. When and if detected, surgery with a curative intent (R0 resection) is associated with a good outcome if there are no liver, peritoneal or distant metastases.

## Competing interests

The author(s) declare that they have no competing interests.

## Authors' contributions

**CM **was the chief surgeon in charge of the case and **VS **was the first surgical assistant; **AB **and **SS **were the pathologists responsible for the histopathological examination, **DB **assisted in the preparation of the manuscript. All authors read and approved the manuscript.
